# Correction: Cervinka, R.; et al. Investigating the Qualities of a Recreational Forest: Findings from the Cross-Sectional Hallerwald Case Study. *Int. J. Environ. Res. Public Health* 2020, *17*, 1676

**DOI:** 10.3390/ijerph18084078

**Published:** 2021-04-13

**Authors:** Renate Cervinka, Markus Schwab, Daniela Haluza

**Affiliations:** 1 University College for Agrarian and Environmental Pedagogy, 1130 Vienna, Austria; renate.cervinka@haup.ac.at (R.C.); markus.schwab@haup.ac.at (M.S.); 2 Department of Environmental Health, Center for Public Health, Medical University of Vienna, 1090 Vienna, Austria

## 1. Error in Table 1

In the original article [1], there was a mistake in Table 1 as published. Values for the places B (Fern Glade) and D (Forest Glade) were interchanged. This resulted from a mistake in editing the table. However, the numbers of the inferential statistics are not affected by it, because they are based on the correct values. Therefore, the interpretation of the results is not affected. Further, the values reported in the original tables (mean, SD) represent values from all available data from the data set. We used listwise deletion for dealing with missing data in the t-tests and RM-ANOVAs. The corrected table reports the standard deviations and means after listwise exclusion. The corrected Table 1 appears below.

## 2. Error in Table 2

In the original article, there was a mistake in Table 2 as published. The post-visit value in the paper published actually represents the pre-visit value. The pre-visit value came from another variable in the SPSS output, and unfortunately we pasted the numbers from this variable. While the inferential statistic is not influenced, because it was based on the values, the effect sizes changed because they were calculated from means and standard deviations given in the original table. Therefore, data in Table 2 must be corrected and the interpretation of the results has to be changed. Accordingly, two sentences need to be changed (see text correction below). The values reported in the original tables (mean, SD) represent values from all available data from the data set. We used listwise deletion for dealing with missing data in the t-tests and RM-ANOVAs. The corrected table reports standard deviations and means after listwise exclusion. The corrected Table 2 appears below.

## 3. Text Correction

The data in Table 2 were corrected and the interpretation of the results was changed. Therefore, the following two sections needed to be changed. First, we corrected 3. Results, Section 3.2, paragraph two: “The most pronounced changes were the increase in perceived restoration, the increase in CF, and the decrease in stress.”

Second, we corrected 4. Discussion, paragraph seven: “Perceived restorative outcome showed the most significant change (increase of 25%) of all measured parameters during the visit. Perceived stress showed the third largest change (decrease of 15%).”

The authors apologize for any inconvenience caused and state that the scientific conclusions are unaffected. The original article has been updated.

## Figures and Tables

**Table 1:** Qualities of the places and the forest.

Scores	(A) Mossy Stones		(B) Fern Glade		(C) Outlook		(D) Forest Glade		(A–D) Total Forest	
	Mean	SD	Mean	SD	Mean	SD	Mean	SD	Mean	SD
Perceived restorativeness potential	7.34 ^C,D^	1.42	7.70 ^C,D^	1.41	6.15 ^A,B^	1.69	6.65 ^A,B^	1.74	6.96	1.11
Vitality	6.09	1.97	6.27	2.04	5.84	2.18	5.78	2.14	6.00	1.63
Widen one´s mind	7.67 ^B,C^	1.40	8.05 ^A,C,D^	1.38	6.69 ^A,B,D^	1.94	7.54 ^B,C^	1.55	7.49	1.16

Note: SD: standard deviation; ^A, B, C, D^ significant post-hoc comparisons (*p* < 0.05, Bonferroni-corrected) for the places (A) Mossy stones, (B) Fern glade, (C) Outlook, and (D) Forest glade.

**Table 2:** Participants’ feelings and perceptions pre- and post-visit.

Scores	Pre-Visit		Post-Visit		*p* ^1^	Effect Size	Change (%)
	Mean	SD	Mean	SD			
Positive affect	59.01	18.83	69.40	17.02	<0.001	0.58 ^a^	−10.39
Negative affect	14.15	18.24	5.20	10.46	<0.001	0.59 ^b^	8.95
Perceived stress	21.28	26.53	6.17	12.52	<0.001	0.69 ^b^	15.11
Perceived restoration	61.05	20.32	85.74	10.66	<0.001	1.5 ^a^	−24.68
Connectedness with nature	60.02	23.59	68.62	22.05	<0.001	0.37 ^a^	−8.60
Connectedness with forest	29.34	26.93	50.35	27.45	<0.001	0.77 ^a^	−21.01
Mindfulness	55.36	15.03	61.89	14.87	<0.001	0.44 ^a^	−6.53

Notes: SD: standard deviation; ^1^ *p* values from t-tests; ^a^ Hedge’s g av (a) and ^b^ Hedge’s g rm (b); POMP-transformed scores.
